# A multicenter analysis of inpatient antibiotic use during the 2015–2019 influenza seasons in the United States: Untapped opportunities for antimicrobial stewardship

**DOI:** 10.1017/ash.2022.265

**Published:** 2022-08-11

**Authors:** Kalvin C. Yu, Vikas Gupta, Heidi Kabler, Janet A. Watts, Amine Amiche

**Affiliations:** 1 Becton, Dickinson and Company, Franklin Lakes, New Jersey, United States; 2 Sanofi, Swiftwater, Pennsylvania, United States; 3 Sanofi, Dubai, United Arab Emirates

## Abstract

Outpatient antibiotic use increases during winter months, but information on temporal changes in inpatient antibiotic use in US hospitals is limited. The use of certain inpatient antibiotics, including extended-spectrum cephalosporins, macrolides, and tetracyclines, was strongly associated with influenza activity during the 2015–2019 viral respiratory seasons.

Bacterial coinfection is common during influenza infections and is associated with increased disease severity in patients with community-acquired pneumonia (CAP).^1,2^ Many patients with influenza-related symptoms, particularly respiratory manifestations, are empirically treated with antibiotics.^3^ Although antibiotic treatment is appropriate in some patients, a recent study found that antibiotic prescribing was unsupported in 79.5% of hospitalized patients with CAP in the United States.^4^

Previous studies have documented increased outpatient antibiotic use during the winter months in which influenza and other respiratory viruses are often prevalent,^3^ but recent evaluations of inpatient antibiotic use (IAU) during influenza season are lacking. IAU patterns can be valuable in informing antimicrobial stewardship policies and strategies. We evaluated changes in IAU in hospitalized adults in the United States over time and potential relationships between IAU and community influenza rates based on data in the BD Insights Research Database.

## Methods

### Study design

We conducted a retrospective, ecological analysis to evaluate possible associations between influenza rates and IAU using real-world data collected between July 1, 2015 and December 31, 2019 from patients ≥18 years old at US acute-care facilities in the BD Insights Research Database (Franklin Lakes, NJ), which includes small and large hospitals with geographical representation across the United States.^5^ Geographic localization was based on US Department of Health & Human Services (HHS) regions. Outcome studies using this retrospective, deidentified data set were approved and informed consent was waived by the New England Institutional Review Board (Wellesley, MA).

The outcome of interest was IAU, calculated as days of therapy (DOT) per 1,000 patient days present for commonly prescribed oral or IV antibiotics, including extended-spectrum cephalosporins (ESCs), β-lactam inhibitor combinations (BLICs), fluoroquinolones (FQs), carbapenems, specified anti–methicillin-resistant *Staphylococcus aureus* (MRSA) drugs (glycopeptides, lipoglycopeptides, and oxazolidinones), lipopeptides, macrolides, tetracyclines, and “other” antibiotics (clindamycin, metronidazole, and aminoglycosides) (see Supplementary Table 1 for specific agents).

Influenza polymerase chain reaction (PCR) and antigen laboratory data were used to determine the influenza positivity rate per 100 tests at inpatient and ambulatory healthcare sites associated with the facility testing laboratory. Influenza testing data therefore represented community influenza rates and were not specific to the inpatient setting. Although other respiratory viruses also display seasonal variations, influenza accounts for most adult cases^6^ and was therefore chosen as the focus of respiratory virus evaluations.

### Statistical analysis

Influenza rates and IAU were calculated for each quarter-year (Q3 2015–Q4 2019) to best encapsulate the concept of a “season.” The sensitivity analyses demonstrated that quarter-years accurately capture seasonal fluctuations in influenza rates. The graphical representations were based on monthly levels to best highlight these seasonal trends.

We used generalized estimated equation models to estimate IAU trends over time and seasonally and the association between IAU and influenza rates. The time-series data were viewed as repeated measures and hospital data were modeled as random effects. Multivariable analyses were performed by antibiotic class while controlling for hospital-level variables (region, bed size, and other characteristics). Beta coefficients indicated the association between variables of interest and were used to evaluate increased IAU for each unit increase in the independent variable; the direction of the association is indicated by positive and negative values. All statistical analyses were conducted using R version 4.0.3 software (R Foundation for Statistical Computing, Vienna, Austria) and the R geepack package. *P* < .05 was considered statistically significant.

## Results

Of 239 acute-care facilities contributing study data, ∼40% (40.6%) were in Health and Human Services (HHS) region 4 (South and Southeast) and 71.6% were in urban locations (Supplementary Table 2). From 2015 to 2019, ESCs, specified anti-MRSA drugs, and BLICs had the highest IAU rates (average of 125, 95, and 94 DOT per 1,000 days present, respectively) (Supplementary Table 3). Data on influenza rates (percentage of positive tests) tracked well with influenza data from the Centers for Disease Control and Prevention.^5,7^

### Trends in IAU

Unadjusted bivariate results showed ascending trends over time (2015–2019) for IAU for ESCs, BLICs, macrolides, and tetracyclines (Supplementary Table 4). In contrast, descending trends were observed in the IAUs of FQs, “other” antibiotics, specified anti-MRSA drugs, carbapenems, and lipopeptides. Seasonal changes in IAU were significant for BLICs, macrolides, tetracyclines, specified anti-MRSA drugs, and lipopeptides.

### Association between IAU and influenza seasons

**Fig. 1.**
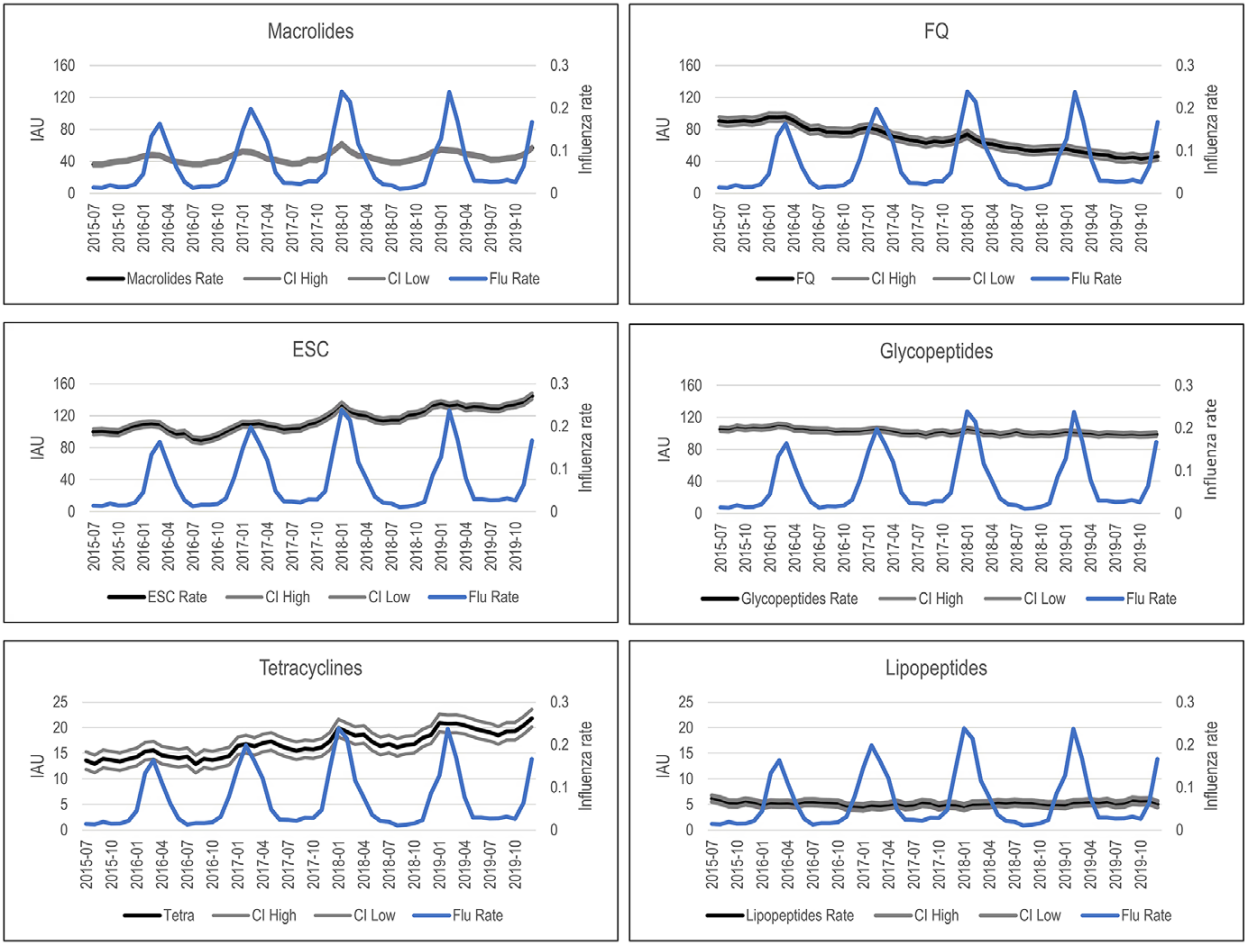
Trends in IAU and influenza rates from 2015 to 2019. *P* < .05 for all associations in multivariate analyses. IAU is expressed in DOT per 1,000 days present (95% CI) and influenza rate is expressed as positive tests per 100. The IAU scale for tetracyclines and lipopeptides has been reduced relative to the other antibiotics to better display variations in these drugs with lower usage. Note. BLIC, β-lactam inhibitor combinations; CI, confidence interval; DOT, days of therapy; ESC, extended-spectrum cephalosporins; FQ, fluoroquinolones; IAU, inpatient antibiotic use; MRSA, methicillin-resistant *Staphylococcus aureus*.

Visually, we observed strong seasonality that matched the influenza season for macrolides, ESCs, FQs, and tetracyclines IAU (Fig. 1). Influenza rates were significantly associated with IAU for macrolides, ESCs, FQs, tetracyclines, and specified anti-MRSA drugs in bivariate analyses (Table 1). In multivariate analyses, associations with influenza rates were retained for all antibiotics with significant results in bivariate analyses, and a significant association between influenza rates and lipopeptide IAU was revealed (Table 1). We detected no associations between influenza rates and IAU for BLIC, carbapenems, or “other” antibiotics.

**Table 1.** Association Between Influenza Rate and Inpatient Antibiotic Use (DOT per 1,000 Patient Days Present)^a^

Antibiotic Class	Bivariate Analyses		Multivariate Analyses	
	Coefficient (CI)	*P* Value	Coefficient (CI)	*P* Value
Macrolides	57.490 (48.417–66.563)	<.001	62.612 (54.192–67.147)	<.001
ESC	7.438 (5.632–8.857)	.006	41.349 (30.592–52.875)	<.001
FQ	21.445 (11.18–31.71)	.001	13.714 (8.030–19.398)	.001
Tetracyclines	9.824 (6.102–13.546)	.008	11.271 (7.889–13.226)	<.001
Specified anti-MRSA drugs	3.368 (0.484–6.252)	.048	8.362 (0.703–16.185)	.042
Lipopeptides	2.420 (−0.513 to 5.353)	.220	2.101 (0.364–4.018)	.027
BLIC	2.055 (−2.150 to 6.210)	.874	1.253 (−0.695 to 3.201)	.688
Carbapenems	1.437 (−1.162 to 4.036)	.850	−0.108 (−1.453 to 0.789)	.841
Other	1.940 (−0.492 to 3.451)	.155	−1.611 (−4.619 to 1.397)	.314

Note. BLIC, β-lactam inhibitor combinations; CI, confidence interval; DOT, days of therapy; ESCs, extended-spectrum cephalosporins; FQs, fluoroquinolones; MRSA, methicillin-resistant *Staphylococcus aureus.*

a Adjusted for hospital geographic region, bed size, urban or rural setting, and teaching or nonteaching status.

## Discussion

Our findings demonstrate clear seasonality of IAU for common antibiotic classes that follows trends in influenza rates. The antibiotic classes showing this association were those often prescribed and recommended for CAP, including ESCs, macrolides, FQs, and tetracyclines. An association with IAU for specified anti-MRSA drugs was also observed. Although in many cases antibiotic use during influenza season may be directed at secondary bacterial infections, inappropriate antibiotic use in hospitalized patients with CAP is common.^4^

We recently reported that antibiotic resistance also demonstrated seasonal trends and was associated with influenza rates.^5^ Our observations on seasonal trends in IAU and antibiotic resistance may highlight an opportunity for improved antimicrobial stewardship efforts during the influenza and viral respiratory season and support the expansion of vaccination programs, a key component of the World Health Organization Action framework on leveraging vaccines to reduce antibiotic use and prevent antibiotic resistance.^8^

In addition to documenting the seasonality of IAU for common antibiotics, our study provides data on trends over time in antibiotic use that update an earlier (2006–2012) study.^9^ The IAU of ESCs, BLICs, macrolides, and tetracyclines continued to increase during the years spanned by our study (2015–2019), and the IAU for FQs decreased. We further observed a decreasing trend in IAU for specified anti-MRSA drugs and carbapenems, a reversal of the increasing trends for these antibiotic classes reported in the earlier study.^9^

Notably, infections with other respiratory viruses, including respiratory syncytial virus and adenovirus, also show seasonal differences that tend to peak around the same time as influenza.^6^ However, these viruses primarily affect children under 5 years of age,^6^ a population not included in this study.

The focus of this ecological study was to evaluate trends in IAU; it was not designed to explore specific infections being treated, clinical outcomes, or appropriate or adequate antimicrobial use. Our study did not include data after 2019 and therefore does not reflect significant changes in antimicrobial treatment patterns in response to severe acute respiratory syndrome-coronavirus-2 (SARS-CoV-2).^10^ It is too soon to know whether SARS-CoV-2 will also demonstrate an influenza-like seasonality, which could further impact IAU trends.

In conclusion, IAU is significantly associated with influenza rates when controlling for geographic region and hospital-level factors. Information on IAU influenced by influenza activity may be useful in guiding infection prevention and control measures, including rapid diagnostic testing and vaccination campaigns for influenza and other respiratory viruses, and in determining policies, strategies, and programs for antimicrobial stewardship efforts to reduce inappropriate IAU during influenza season.

## Supplementary material

For supplementary material accompanying this paper visit https://doi.org/10.1017/ash.2022.265. click here to view supplementary material
