# Supplementary material for: A multicenter analysis of inpatient antibiotic use during the 2015–2019 influenza seasons in the United States: Untapped opportunities for antimicrobial stewardship
Source: Antimicrob Steward Healthc Epidemiol. 2022 Aug 11;2(1):e140. doi: 10.1017/ash.2022.265 (PMC9726518; doi:10.1017/ash.2022.265)
Supplement: Supplementary file 1 [file S2732494X22002650sup001.docx]

**A Multicenter Analysis of Inpatient Antibiotic Use During the 2015-2019 Influenza Seasons in the US: Untapped Opportunities for Antimicrobial Stewardship**

Kalvin C. Yu MD, Vikas Gupta PharmD, Heidi Kabler MD, Janet A. Watts PhD, Amine Amiche PhD

**SUPPLEMENTARY MATERIAL**

**Supplementary Table 1. Antibiotic Classes and Agents Included in Inpatient Antibiotic Use Analyses**

| **Antibiotic class** | **Agents** |
| --- | --- |
| Extended-spectrum cephalosporins | Cefotaxime, ceftazidime, ceftriaxone, cefepime, ceftaroline, ceftazidime-avibactam, ceftolozane/tazobactam |
| β-lactam inhibitor combinations | Piperacillin/tazobactam, ampicillin/sulbactam, amoxicillin/clavulanate |
| Fluoroquinolones | Levofloxacin, moxifloxacin, norfloxacin, ciprofloxacin, ofloxacin, delafloxacin |
| Carbapenems | Meropenem, imipenem/cilastatin, imipenem/cilastatin//relabactam, ertapenem, doripenem, meropenem/vaborbactam |
| Specified anti-MRSA drugs | Vancomycin, oritavancin, dalbavancin, telavancin, linezolid/tedizolid |
| Lipopeptide | Daptomycin |
| Macrolides | Erythromycin, azithromycin, clarithromycin |
| Tetracyclines | Doxycycline, tigecycline, minocycline, omadacycline, eravacycline |
| Others | Clindamycin, metronidazole, aminoglycosides (gentamicin, tobramycin, amikacin, and plazomicin) |

**Supplementary Table 2. Hospital Characteristics**

| **Characteristic** | **n** | **%** |
| --- | --- | --- |
| Overall | 239 |  |
| Urban/rural |  |  |
| Urban | 171 | 71.6 |
| Rural | 68 | 28.5 |
| Teaching status |  |  |
| Non-teaching | 200 | 83.7 |
| Teaching | 39 | 16.3 |
| Bed size |  |  |
| < 100 | 67 | 28.0 |
| 100-300 | 92 | 38.5 |
| > 300 | 80 | 33.5 |
| HHS Region |  |  |
| Region1 (CT, ME, MA, NH, RI, VT) | 13 | 5.4 |
| Region 2 (NJ, NY) | 3 | 1.3 |
| Region 3 (DE, DC, MD, PA, VA, WV) | 10 | 4.2 |
| Region 4 (AL, FL, GA, KY, MS, NC, SC, TN) | 97 | 40.6 |
| Region 5 (IL, IN, MI, MN, OH, WI) | 32 | 13.4 |
| Region 6 (AR, LA, NM, OK, TX) | 32 | 13.4 |
| Region 7 (IA, KS, MO, NE) | 16 | 6.7 |
| Region 8 (CO, MT, ND, SD, UT, WY) | 11 | 4.6 |
| Region 9 (AZ, CA, HI, NV) | 23 | 9.6 |
| Region 10 (AK, ID, OR, WA) | 2 | 0.8 |

**Supplementary Table 3. Mean Inpatient Antibiotic Use (DOT/1000 Patient Days Present [SD]) Between 2015 and 2019**

| **Antibiotic class** | **Year (number of facilities)** | | | | | |
| --- | --- | --- | --- | --- | --- | --- |
|  | **2015**  **(n=98)** | **2016**  **(n=145)** | **2017**  **(n=173)** | **2018**  **(n=207)** | **2019**  **(n=231)** | **All years**  **(n=239)** |
| DOT/1000 patient days present (SD) | | | | | | |
| ESC | 107.38 (49.44) | 107.92 (52.87) | 119.41 (57.58) | 128.88 (57.49) | 141.00 (62.12) | 124.96 (58.89) |
| Specified anti-MRSA drugs | 97.56 (34.06) | 98.02 (37.45) | 96.56 (37.83) | 93.22 (38.43) | 94.01 (43.52) | 95.34 (39.40) |
| BLIC | 82.79 (37.88) | 95.41 (39.56) | 96.53 (40.47) | 93.94 (40.76) | 94.99 (44.33) | 94.32 (41.45) |
| Macrolides | 47.05 (42.63) | 48.87 (31.21) | 53.99 (43.59) | 54.22 (34.21) | 57.00 (31.96) | 53.51 (36.19) |
| FQ | 109.19 (51.59) | 99.49 (56.50) | 82.02 (44.45) | 68.40 (41.42) | 60.33 (49.47) | 77.51 (50.70) |
| Carbapenems | 33.23 (26.41) | 30.69 (19.73) | 27.93 (21.09) | 24.78 (19.46) | 24.91 (20.33) | 27.15 (20.83) |
| Tetracyclines | 13.43 (11.75) | 14.38 (13.27) | 16.43 (14.76) | 18.25 (16.81) | 21.20 (19.75) | 17.65 (16.63 |
| Lipopeptide | 3.89 (4.45) | 3.66 (4.36) | 4.04 (9.42) | 3.54 (6.12) | 3.95 (8.16) | 3.81 (7.26) |
| Other | 80.26 (26.05) | 72.99 (26.29) | 70.21 (27.40) | 63.11 (26.26) | 58.79 (26.12) | 66.41 (27.27) |

Note: BLIC, β-lactam inhibitor combinations; DOT, days of therapy; ESC, extended-spectrum cephalosporins; FQ, fluoroquinolones; MRSA, methicillin-resistant *Staphylococcus aureus*; SD, standard deviation.

**Supplementary Table 4. Model Detected Trends in Inpatient Antibiotic Use (DOT/1000 Patient Days Present [95% CI]) Over Time and Seasonally from 2015-2019**

Note: BLIC, β-lactam inhibitor combinations; CI, confidence interval; DOT, days of therapy; ESC, extended-spectrum cephalosporins; FQ, fluoroquinolones; IAU, inpatient antibiotic use; MRSA, methicillin-resistant *Staphylococcus aureus*; Q, quarter.

| Antibiotic class | Beta coefficients | | | |
| --- | --- | --- | --- | --- |
|  | Over time  (2015-2019) | P-value | Seasonally  (Q1 higher,  Q3 lower) | P value |
| Antibiotics with increasing IAU over time | | | | |
| ESC | 11.002 (6.727-15.277) | 0.005 | 1.027 (-0.131-2.185) | 0.088 |
| BLIC | 3.772 (2.413-5.131) | 0.003 | 0.910 (0.394-1.426) | 0.042 |
| Macrolides | 1.500 (0.911-2.089) | 0.005 | 1.134 (0.566-1.702) | 0.023 |
| Tetracyclines | 0.290 (0.185-0.395) | 0.003 | 0.092 (0.002-0.182) | 0.023 |
| Antibiotics with decreasing IAU over time | | | | |
| FQ | -10.881 (13.643-8.119) | <0.001 | 0.0755 (-0.209-1.041) | 0.396 |
| Other | -2.060 (3.183-0.937) | <0.001 | 0.048 (-0.118-0.221) | 0.283 |
| Specified anti-MRSA drugs | -1.052 (1.675-0.429) | 0.031 | 1.883 (0.873-2.893) | 0.046 |
| Carbapenems | -0.908 (1.347-0.469) | 0.019 | 0.593 (0.194 -0.992) | 0.069 |
| Lipopeptides | -0.215 (0.327-0.103) | 0.022 | 0.015 (0.006-0.024) | 0.046 |
